# The Learning Collaboratory: developing and evaluating public health students’ skills while promoting community health

**DOI:** 10.3389/fpubh.2023.1269840

**Published:** 2023-11-20

**Authors:** Viviana E. Horigian, Tatiana Perrino, Julie Kornfeld, Renae D. Schmidt, Sophia T. Gonzalez

**Affiliations:** ^1^Department of Public Health Sciences, University of Miami Miller School of Medicine, Miami, FL, United States; ^2^School of Nursing & Health Studies, University of Miami, Coral Gables, FL, United States; ^3^Mailman School of Public Health, Columbia University, New York, NY, United States

**Keywords:** experiential learning, community health, integrative learning experience, public health education, service learning

## Abstract

**Introduction:**

Complex and continuous developments in health and healthcare require innovative changes in programs that educate public health scientists and professionals. Public health change agents need critical competencies to confront today and tomorrow’s leading problems including leadership, communication, interprofessional practice, and systems thinking.

**The context: challenges in public health education:**

Public Health training programs teach competencies through their applied field experience and culminating project, typically late in the program, and often implemented in isolation from peers and faculty. Objectives and skills do not always align closely with community-based program needs. Students pursuing a degree in science in public health need to deeply comprehend multi-dimensional and interconnected systemic problems and communicate with diverse stakeholders across disciplines to produce relevant community-engaged research. The University of Miami Public Health Learning Collaboratory (LC) was established to transform the learning experience of public health master’s students by providing opportunities to develop necessary core skills for effective public health practice early in their training, while applying these skills to address real-world public health needs in the community.

**The Learning Collaboratory: structure, pedagogical approach and programmatic details:**

Spanning an average of 3 semesters, the LC promotes student involvement in collaborative and impactful capstone and thesis projects. Practice-based teaching and service learning are central approaches to teaching cross-cutting competencies of leadership, communication, problem solving, collaboration, and systems thinking in public health. Significant to the approach is the engagement of previous cohorts of senior students to teach back to junior students, further integrating concepts learned. Long term alumni feedback recognized strengths of the program, including its structure, teamwork & collaboration, critical thinking & problem solving, guidance, nurture & support, teaching back, and content & curriculum. Community partners agreed the LC prepared students to practice in the field of public health.

**Discussion:**

The LC is a promising model for master’s level public health education and community application, given the opportunities it provides to strengthen and integrate students’ public health skills in a supportive environment, and enhance the transferability and sustainability of student and faculty’s community public health work.

## Introduction

Addressing continuous and complex challenges in public health and healthcare requires innovative approaches and well-prepared workers and scientists (1–6). To respond to challenges, such as rising health care costs and health disparities, public health education must provide a strong foundation in substantive and methodological areas, but also opportunities to strengthen cross-cutting professional competencies such as effective communication, community and cross-disciplinary collaboration, problem-solving, and leadership. Towards this end, over the years, several reports and guidance documents have been written about the significance and urgency of building professional competencies among public health students to prepare the change agents needed to confront the public health problems of today and the future (1, 2, 4, 7). In addition, the importance of interprofessional education and collaborative practice has been heightened by major organizations as instrumental to bringing about improvement in health for persons and communities. In 2021, the Council on Education in Public Health revised accreditation criteria for public health programs that have redefined the foundational knowledge and competencies for Master of Public Health (MPH) degrees. These criteria group foundational competencies across professional development domains, including leadership, communication, inter-professional practice and systems thinking (8). The culminating experience, defined under the new criteria as “applied practice experience” and “integrative learning experience,” continues to be seen as a program’s central educational component intended to strengthen the student’s area of concentration and provide opportunities to learn and apply analytic, synthesis and evaluation skills (6, 9).

These cross-cutting professional skills are equally important for public health scientists, including Master of Science (MS) in public health students. For scientific findings to be influential in ultimately solving complex public health problems, students must be able to deeply comprehend multi-dimensional and interconnected systemic problems, communicate with diverse stakeholders and scientists across disciplines, and participate in “team science,” that is “…research conducted by more than one individual in an interdependent fashion…” (5). This kind of research can generate more innovative, robust and influential science, but it is challenging to accomplish. It requires well-honed professional skills such as effective communication, community and cross-disciplinary collaboration, problem-solving, and leadership are fundamental.

### Context: The challenge in public health education

Public health master training programs have been challenged to innovate their educational approaches to help students gain foundational competencies in public health and prepare students for the workforce. While traditional classroom courses play an important role in teaching public health concepts and may offer the chance to apply this knowledge, they might not routinely provide opportunities to practice these cross-cutting skills in real-life settings. Many training programs tackle the culminating experience (i.e., capstone, thesis) as the vehicle by which students obtain these competencies and offer these integrative learning opportunities. However, under this approach, students might begin their culminating experience late in the program, might implement it in isolation from peers and faculty, and partners in the community might be challenged with students arriving one at a time, with skills that might not align closely with community-based program needs. These limitations in approach were recognized by master’s program advisors at the Department of Public Health Sciences, University of Miami. First, students began their culminating experiences late in the program, reducing opportunities for learning and ensuring these were impactful. Second, while students become immersed in community work during their capstones or considered the long-term impact of their thesis, they were frequently isolated from the master’s program and fellow students, reducing valuable opportunities to integrate academic and practical work and to collaborate with peers and faculty. Finally, the public health work they conducted was often time-limited and discontinuous, given that the work often ended before goals were achieved. For example, a student might conduct an insightful needs assessment that identified high rates of substance abuse in a neighborhood and recommend strategies to address this, but the student did not have time to implement these strategies by the completion of their culminating experience. This discontinuity limited the utility of student’s work and the service provided to surrounding communities, many of which have significant health needs. To address these shortcomings, the University of Miami Public Health Learning Collaboratory (LC) was established in 2014 as an educational initiative to transform the learning experience of master’s students in public health by providing opportunities to develop core skills necessary for effective public health science and practice, and simultaneously apply these skills to address real-world public health needs. The objective of this –case study is to describe the structure and organization of the LC, its pedagogical framework, and to present an evaluation of the long-term results.

### The Learning Collaboratory: Structure, pedagogical approach and programmatic details

The LC’s teaching strategies are aligned with adult learning theories and models, emphasizing real application of concepts as well as experiential, practice based and service learning (10–12). Spanning an average of 3 semesters, the LC promotes student involvement in collaborative and impactful capstone and thesis projects. Students begin the LC during their first semester in the public health program by enrolling in a 3-credit course (Fall), alongside other new students. This initial course is comprised of small groups of students and community partners around common thematic areas of interest, such as Access to Health Care, Health in Latin America, Prevention with Children and Families, and HIV and Substance Use. Students are selected to join the course based on 1) demonstrating a professional interest in the group topics, 2) their understanding of the substantive area and LC goals, and 3) committing to participate for three semesters. Both in-class sessions and field visits blend conceptual and applied learning in the community.

In-class sessions include seminars, workshops, and group discussions. With the full class and within their small groups, students learn and practice concepts fundamental for public health science and change, including evidence based public health, community engagement, needs assessment, logic models and models of change, program planning and evaluation, communication, and ethics, among others. Through discussions, interactive exercises and home-learning, students enhance their understanding of substantive areas (e.g., epidemiology of diseases, determinants of health). As students learn class concepts, each thematic group meets with pre-selected community agencies to apply what they have learned and to more deeply understand the complexity of existing public health problems from diverse professionals working to address these problems. The students’ applied work aims to understand the community and agency’s needs, with field visits involving community meetings with partners, stakeholders, and instructors. Community representatives become active partners in the LC, with the projects and theses emphasizing community-based participatory research methods that engage key community stakeholders throughout the life of the project (13).

Modeling is a central part in the learning process. Instructors lead initial community sessions to demonstrate characteristics of effective engagement, such as active listening, effective communication, promoting participation and engagement of partners, identifying evidence-based programs that can help communities frame their approach to addressing public health problems. Students initially observe and subsequently become active participants in these community sessions. During community visits, students engage with agencies, residents, and other professionals around public health needs, as well as practice the key professional competencies introduced in class, such as communication, problem-solving, and building inter-disciplinary and community collaborations. Targeted assignments evaluate students’ knowledge and skills, such as illustrating a project’s logic model or writing a needs assessment. Students finish their first semester having a proposed concentration, a deep understanding of the public health problem, including a robust review of the literature and evidence base. Students also finish this initial semester with a strong relationship with the community agency, as well as other professionals and stakeholders in their area of public health interest. MPH students finish with an established field site placement for their capstone.

During the second semester (Spring), MPH students begin their 150-h capstone field experience (i.e., applied practice experience). They draft learning objectives and activities for the field experience, individualized to meet the student, agency, and community’s needs. For example, to address low rates of community HPV vaccination, a student interested in prevention of sexually transmitted infections worked with a mobile pediatric clinic to analyze existing data on acceptability and completion of HPV vaccine among adolescent patients. While embedded in the community, students apply and reflect upon what they have learned in class. MS students, on the other hand, work on their master’s thesis proposal, which is also informed by their connections with community stakeholders and other professionals. During this second semester, students meet five times with the instructors of the LC, with the goal of reflecting on the field experience and thesis proposal as these unfold, trouble shooting and receiving guidance as they work on these products.

The focus on practice-based teaching and service learning are central approaches to teaching these cross-cutting skills in public health (14–16). These approaches provide a pragmatic and progressive learning experience while meeting societal needs (17). The problem-based learning approach further allows the specific public health problems and challenges experienced in community to provide the groundwork for developing students’ needed to competently solve public health problems. Notably, these community-immersed, practice-based experiences place students in interprofessional settings, where critical competencies of interprofessional education are exercised. As students provide service to the community by supporting ongoing projects, they plan their capstone projects and theses, as informed by the community, to ensure projects are relevant and impactful. Projects and theses are executed in subsequent semesters, typically concluding in final semester.

**Figure 1:**
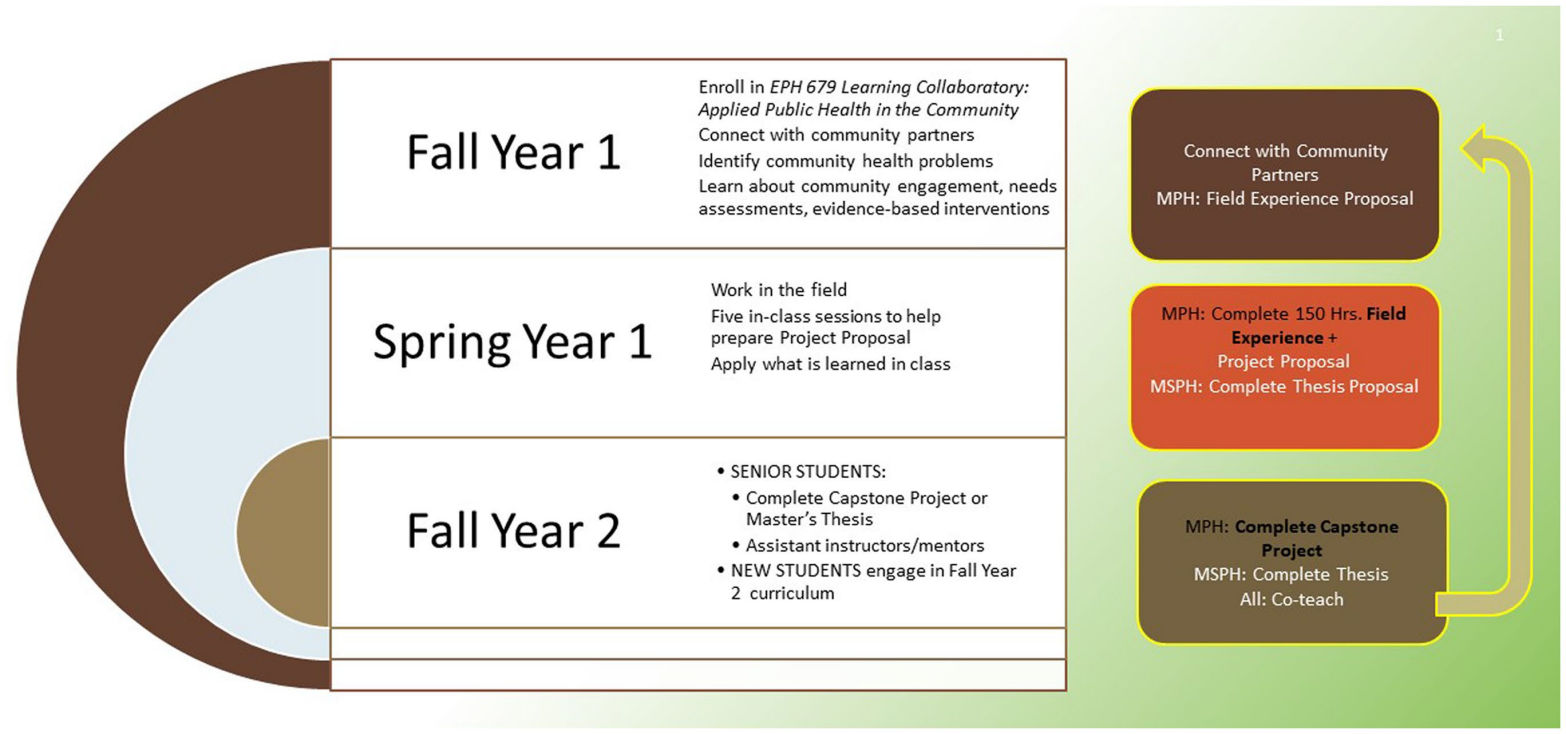
Timeline and structure of the Learning Collaboratory.

The final semester (Fall) is when student integration of knowledge and skills is most prominent. These senior-level students work on their projects and present their insights to the incoming cohort of LC students. The senior-level students are invited to help teach the incoming cohort of students on topics they have learned and implemented, for example community stakeholder engagement or culturally-informed interventions. Similar to what Kolb (10) describes, students have concrete experiences in the field, which provide the foundation for reflective observation when they have the opportunity to consider what is working or failing. This reflection promotes thinking about ways to improve during the next attempt, a form of abstract conceptualization, and the “*teach back”* facilitates integration of concepts learned (18, 19). Overall, this three-semester sequence permits each student cohort to complete their capstones and help train and mentor students from the incoming cohort, fostering the development of new students into senior-level students better prepared to become public health scientists and practitioners. Figure 1 illustrates the model by steps and semesters.

Feasibility of the multi-semester educational initiative has been examined from the program’s start. Preliminary results on feasibility, acceptability, and comparison of the LC MPH students with a cohort of students pursuing the traditional approach to the field experience and capstone project were presented at the American Public Health Association Conference, 2015 (20). LC students showed improved leadership, communication, and cultural competence skills as compared to control students. While these differences were not statistically significant, the LC students showed a significantly better sense of classroom community. Subsequent evaluations of the program revealed strong satisfaction and LC students repeatedly reported that the program had enhanced their knowledge, that they have enjoyed it, would recommend it to fellow students, had learned about teamwork and communication skills, and believed the LC had made them more competitive for the job market. While initial acceptability and early results were reassuring, evaluations were not completed at program exit, not allowing for student reflections on their use of the skills on the job, and further limited by small number in the cohorts.

To assess longer term results of the LC, during the months of March and April 2023, a Qualtrics survey was conducted with alumni of the LC cohorts admitted from Fall 2014–2021 and with community partners of the LC. MPH students participated in all LC cohorts beginning in 2014, while Master of Science in Public Health (MSPH) students and MS in Prevention Science & Community Health students participated beginning in 2019. Each of 55 LC alumni with recorded contact information, as well as five longstanding community partners of the LC, received an email invitation to complete the online survey. Upon survey completion, participants were directed to a separate, unlinked Qualtrics survey to input their email to receive a $35 Amazon e-gift card as compensation for their time and feedback. As this study captured participant feedback regarding the program itself, it was determined by the University of Miami Institutional Review Board to not require IRB approval.

The alumni survey began with two descriptive questions, including degree pursued while in the program (MPH, MSPH, or MS in Prevention Science) and current employment type. Next, alumni were asked to rate, on a 5-point Likert scale from *Not at all helpful* to *Extremely helpful,* the extent which the LC was helpful to their development each of five public health critical skills: Critical Thinking, Communication, Problem Solving, Collaboration, and Leadership. Then, using the same Likert scale, alumni were asked to reflect on: 1) the extent to which applying skills learned in class to address real world public health problems with community partners helped them develop understanding and/or skills in community collaboration and community engagement, 2) the extent which the teach-back component helped them to integrate concepts, and 3) the extent which the teach-back component was helpful to their professional development. As in first studies on feasibility, acceptability, and comparison between LC students and students pursuing the traditional approach, survey items were developed by two authors (VEH and TP), focusing on the competencies developed by the LC but adapted to capture the long-term reflection on the experience. As part of the survey, alumni were also invited to respond to a series of three open-ended questions describing the strengths of the program, the weaknesses of the program, and any additional reflections they would like to share.

In their survey, community partners were asked to rate, on a 5-point Likert scale from *Strongly Disagree* to *Strongly Agree* (or Not Applicable), the extent to which LC students were adequately prepared for each of 25 core public health skills. These questions are the standard questions used in the University of Miami Department of Public Health Sciences to obtain feedback from organizations where students’ intern. Some of the skills assessed included an evaluation on students’ ability to use concepts of behavioral sciences to analyze and solve public health problems, interpret health information from local, and state level, determine program needs and rationale for operation, demonstrate professionalism, and skills and competencies needed to enter the public health profession. The survey similarly ended with three open-ended questions where participants were asked to describe the strengths of the program, the weaknesses of the program, and any additional reflections they would like to share.

Frequencies and percentages of responses were calculated for each Likert-scale item. For open-ended questions, three authors (VEH, RDS, STG), using a qualitative inductive approach, reviewed each of the responses to identify core themes emerging from the data and came to a consensus on the final themes and definitions before they coded each response as representative of a given theme or not. Frequencies and percentages of identified themes were then calculated. Cronbach’s alpha coefficient was calculated to assess the internal consistency of the quantitative survey items.

## Results

### Alumni responses – quantitative

Thirty alumni responded to the survey (response rate 54.5% of all LC alumni). Among them, 66.7% were in the MPH program, 26.7% in the MSPH program, and 6.6% in the MS in Prevention Science and Community Health program. When asked about their current employment type, almost half (46.7%) were working for an academic institution, 26.7% for a non-profit organization, 13.3% for government and for community organization, and 10% for private sector. There were also 10% of alumni enrolled in graduate studies and 6.6% who were pre- or post-doctoral scholars. One alumnus selected “other” and reported that they were working in the pharmaceutical industry (Note: respondents could choose more than one employment type).

When asked to rate the extent to which the LC helped their development public health competencies, over three-quarters of alumni responded *Very helpful* or *Extremely helpful* to each of the five competencies, including Critical Thinking (83.3%), Communication (80.0%), Problem Solving (76.6%), Collaboration (86.6%), and Leadership (83.3%). Similarly, 83.3% of alumni responded *Very helpful* or *Extremely helpful* to the extent to which they developed understanding and/or skills in community collaboration and community engagement and to the extent to which teach-back helped them to integrate concepts. 73.4% responded *Very helpful* or *Extremely helpful* to the extent which teach-back was helpful to their professional development. The internal consistency of survey items was acceptable with a Cronbach’s α of 0.95 (Tables 1, 2).

**Table 1:** Characteristics of alumni respondents.

		*N*	%
All		30	100
Program	MPH	20	66.70%
	MSPH	8	26.70%
	MS, Prevention Science	2	6.60%
Employment type*	Private sector	3	10%
	Government	4	13.30%
	Academic Institution	14	46.70%
	Community based organization	4	13.30%
	Non-profit	8	26.70%
	Pre- or Post-doctoral scholar	2	6.60%
	Enrolled in graduate studies	3	10%
	Not currently employed	0	0
	Other	1	3.33%

*Some participants had more than one employment type.

**Table 2:** Frequencies and percentages of alumni ranking of extent to which LC helped them develop public health competencies.

		Not at all helpful	Not so helpful	Somewhat helpful	Very helpful	Extremely helpful
To what extent was the Learning Collaboratory helpful to your development each of the following competencies:	Critical thinking	0	1 (3.3%)	4 (13.3%)	12 (40%)	13 (43.3%)
	Communication	1 (3.3%)	0	5 (16.7%)	7 (23.3%)	17 (56.7%)
	Problem solving	1 (3.3%)	2 (6.6%)	4 (13.3%)	10 (33.3%)	13 (43.3%)
	Collaboration	0	2 (6.6%)	2 (6.6%)	10 (33.3%)	16 (53.3%)
	Leadership	1 (3.3%)	1 (3.3%)	3 (10%)	12 (40%)	13 (43.3%)
Through partnerships with public health practitioners, students address real-world public health needs in the community by applying essential skills they learn in class. To what extent do you think this component helped you develop understanding and/or skills in community collaboration and community engagement?		1 (3.3%)	2 (6.6%)	2 (6.6%)	10 (33.3%)	15 (50%)
To what extent do you think the teach-back component helped you to integrate concepts (e.g., reflect on topics and concepts you had learned, in a way that it helped expand your understanding)?		2 (6.6%)	1 (3.3%)	2 (6.6%)	9 (30%)	16 (53.3%)
To what extent do you think the teach-back component was helpful to your professional development (e.g., strengthened skills to succeed in the workplace and in your profession)?		1 (3.3%)	4 (13.3%)	3 (10%)	8 (26.7%)	14 (46.7%)

### Community partner responses – quantitative

Three community partners responded (response rate 60%). Partners *Agreed* that students of the LC demonstrated their preparation to practice in the field of public health (75%), produced products that were professional and appropriate (100%), and demonstrated the competencies required for their public health degrees (75%). They *Strongly Agreed* (75%) or *Agreed* (25%) that students accepted responsibility and fulfilled commitments to the agency, *Strongly Agreed* (75%) or *Agreed* (25%) that students were adaptable and worked well with agency staff and clients or citizens served by the agency, as well as *Agreed* or *Strongly Agreed* (75%) that students were able to assess results of their programs and make recommendations based on public health concepts. Community partners *Agreed* (100%) that students produced projects that were beneficial to the program or agency.

### Alumni responses – qualitative

After initial review of the data, six themes emerged as strengths of the LC. *Structure* was an identified strength mentioned in 21 of 29 (72.4%) of responses provided. Structure was defined as overall organization duration over three semesters, set and established partners, sequence and nature of topics to prepare students for field experience and capstone. Highlights of comments regarding structure were: *“The hands-on mentorship and feedback throughout the community partnership component was invaluable. These relationships can be tricky to navigate, but the support and structure provided by the LC helped me thrive in making connections, understanding roles, delineating objectives, and pulling off my final project,” “I appreciated getting off to an early start on Field Experience components as I was able to utilize so much more of my time enrolled in the MPH program towards building meaningful partnership and deliverables.”* Other comments under this theme were: “*… it also kept me on track with really planning out my thesis and figuring out exactly what pieces were required along the way. I was not aware of how much time and trial and error even finding a feasible thesis project could take, and I’m glad I started making strides early with this class.”*

*Teamwork & Collaboration* was defined as relationships with other students and with partners, something that was identified as a strength in 15 of the responses (51.7%). Some alumni comments highlighting this theme were: “*Helped me build connections with leaders in public health and explore* var*ious public health PH opportunities. This helped target my research questions and I was able to network with stakeholders. I also was able to envision a career in PH through the collaborative and connect with my peers and those in the cohort below me,” “A small group that allowed us collaborate on our projects and interests,”* and that it *“created stronger bonds and friendships within the group.”*

*Critical Thinking & Problem Solving –* defined as the ability to think critically and analyze problems, determine their potential causes and explore solutions – was found as a strength in 4 responses (15.8%), represented by comments such as: “The *strength of that class was the importance that gave to critical thinking and different methods of problems solving,”* *… the class challenged us to think about the ‘why’ and try to understand things that are not at the surface level. As we challenge to think about the why, I felt we were empowered to reach out to the community and make an impactful change.”*

*Guidance, Nurture & Support –* defined as mentorship and guidance by instructors, sustained support, and feedback – was identified as a strength in 10 (34.5%) of the responses. Participants stated “Both *Dr. H and Dr. P* [LC instructors] *are amazing in their own ways and together they made such a great team of mentors. It was so helpful to have the guided first experience into the research-community world,” I believe I benefitted from the planning and guidance available to me so much earlier than my peers,” “I felt so much more supported.”*

*Teaching Back –* defined as senior students co-teaching concepts to incoming cohorts – was identified as a strength in 3 of the responses (10.3%). Some of the comments shared were: “*As a first year, hearing other students’ experiences, lessons learned, and approaches helped me gain a broader understanding of competencies in* var*ious scenarios. Then returning to share my experiences and “teach” around these competencies was the icing on the cake - it made me feel empowered and validated,” “Having chances to present to the cohort and to the cohorts afterwards was great for practicing presenting parts of the thesis, and I found it very helpful to do things towards the project in a bite-sized amounts.” Content & Curriculum* was identified as a distinct theme as well and identified as a strength in 9 (31%) of responses, highlighted by comments such as: “*Setting the foundation of core classes/ competencies for a future Public Health Professional. I feel it prepared me very well for the real world,” “The professors ingrained in me the idea of writing a very good thesis proposal and they were correct in that it laid a wonderful foundation that carried me through the thesis writing process,”* “*Learned leadership skills and improved my public speaking and communication with my mentors.”*

To the question about weaknesses, most of those responding indicated there were no weaknesses that they could think of. After a review of the data, six themes/categories emerged for weaknesses. Among those noting a weakness, the most common was *Need for more Flexibility* identified in 4 (13.8%) of responses. Comments in this theme included “*Sometimes the community partner was not a great match or fit, but that is not unlike real-life,” “My only suggestion is to give us more time between deadlines.” Premature Exposure of Concepts* – defined as students feeling inadequately prepared for course components – w as identified in 3 (10%) of the responses. Sample comments included: “*I remember being very overwhelmed with having to come up with a proposal my first semester in the program,” “The exercise in writing the proposal for the thesis or capstone wasn’t as helpful because it was too earlier for many of us to know what we would be doing.” More Time Needed for Teaching –* defined as needing more time from instructors- was identified as a weakness in 3 (10%) of responses. Comments included statements such as: *“I would consider insufficient time of teaching for this magnific class as weakness,” “I believe the Collaboratory is what the students make out of it…. Otherwise, the lectures are probably the next-weakest point of the program, but it’s hard to compare a priori learning of generally useful concepts to practical, hands-on experience specific to my area of interest.” Insufficient Skill Development –* defined as requiring more academic support within larger master’s programs, having gained insufficient experience, or preparation for job placement – was identified by 5 responses (17%). Comments under this theme included: “*Not enough readily available academic support resources through the program. For instance, if I did not understand a certain topic or concept in one of my classes I felt like I did not have much to support to lean on for help,” “No job placements or alumni presentations,”* “*It’s harder to get an in-depth understanding of the individual concepts, I felt like the semester went by so quickly that I did not get to master all of the concepts.” Program might not be Best for MSPH Students* was identified as weakness theme and coded in 4 (13.8%) responses. Comments included: “*I think it could be valuable if there was a better way to integrate community engagement with the thesis project. At times it felt like community engagement projects in the Learning Collaboratory were directed toward the capstone,” “Also, the program may not have been as well suited for some MSPH students,” “I cannot think of many weaknesses of the program. I do however remember being initially confused on where it was possible or feasible to get a data set on a project.”* Finally, *Less than an Ideal Collaboration with Partner* emerged as a weakness theme and coded in 6 (21%) of the responses. Students identifying this indicated that: *“I heard from some during the program that they felt a lack of support or engagement from their community preceptor and/or mentor/advisor, which led to delayed timelines and mediocre final projects,” “Not all community partners are as responsive/able to assist students in completing projects or meeting requirements in a timely manner,”* and finally *“Accountability on the partner side”* was named as a weakness.

### Community partner responses – qualitative

Identified strengths included faculty support, motivation and preparation: *“The support of faculty, some classes prepare them for the experience while there are opportunities for improvement,” “For the most part, students are motivated and well-prepared. They are able to analyze needs and gaps from a systems level approach and public health perspective. They are an asset to [center’s] interdisciplinary perspective.”* While one partner identified no weaknesses, others identified certain weaknesses in students which included statistical skills: *“Some students lack skills necessary to carry out their projects-especially in the area of statistics, implementation and program evaluation concepts,” “A potential solution is to have a better connection between the advisor and the community partner.”* An additional comment was: *“working with the Collaboratory has been a worthwhile experience.”*

## Discussion

Alumni of the LC cohorts 2014–2021 found that the LC helped their development in public health competencies including Critical Thinking, Communication, Problem Solving, Collaboration and Leadership. Importantly, they felt that the LC helped them understand and value the concept of collaboration and they valued the “teach back” approach as one that expanded understanding and helped in professional development. Community Partners of the LC agreed that students produced projects that were valuable to their organizations. Qualitative analyzes revealed strengths and weaknesses. Structure, Teamwork & Collaboration, Critical Thinking & Problem Solving, Guidance, Nurture & Support, “Teaching Back,” and Curriculum & Content were found to be strengths of the LC. While several alumni did not identify any weaknesses, some recommended more flexibility, and more time for teaching. Others recommended revisiting the inclusion of the MS programs in ways to more fully align it to their needs, addressing potentially premature exposure to certain concepts, and finding ways to address student-partner fit. Indeed, teaching team science skills and ensuring that MS students fully utilize and apply these relationships in their thesis projects may require additional time and effort, as has been clear in the science of team science literature (5). Partners found that the experience was overall valuable but additional areas of improvement could include stronger preparation on methodology, as well as check-ins with advisors. While the two LC instructors were advisors to most MPH and MS students, as the LC program grew, students were sometimes assigned outside advisors. This may have reduced the community agency’s connection to the faculty, which can be important.

Practice-based teaching and problem-based learning have been recognized as a pedagogical approach that can ground professional public health skills education, as it provides applied educational opportunities for public health students (16). For instance, Greece, Wolff and McGrath (15) have described a conceptual model of practice-based education for MPH students, STEPS, which include securing partnerships, training and technology, engagement and implementation, presenting deliverables, and sizing up for results. The model ensures that students focus on learning through addressing existing problems and needs, as well as the design and the implementation of relevant deliverables in a public health agency. The capstone or culminating project resulting from the field experience is where the students produce a concrete deliverable for the community partner. Other models for teaching public health students in community work have embraced the pedagogy of collegiality. As described by Turalba and Malik (15), the essential features of this pedagogy incorporate experiential learning and critical thinking skills but inherent to its execution are principles of collaboration guided by principles of listening, relationship and community building, valuing diversity and collaboration, which are critical in team science and applied public health. Linnan et al. (14) highlight principles of Group Based Service Learning approaches, including cultivating authentic partnerships and planning projects with partners, giving students choices in selecting projects, providing a mentoring team, establishing intentional structures and processes to promote the partnership and encourage reflections, and a culminating event that celebrates the accomplishments. The LC includes several of these components, such as securing the partnership by offering students an array of pre-set partners, supporting the students in their exploration of the objectives and activities to be pursued in the field and helping assess together with the partner their relevance and feasibility for execution. It also supports technical training by exposing students to the content “tool kit” necessary for field work and providing ample feedback and mentorship along through structured time. Finally, the LC promotes reflection and supports teach back as means of integrating concepts learned, but most importantly to identify potential next steps for incoming cohorts of students in the field to support sustainability of the work of community partners.

Several lessons have been learned to date that could help future applications of the LC. Critical to success is the commitment of students to a three-semester trajectory. Additionally, students have to be ready to embrace community organizations’ public health priorities with a selfless stance while committed to achieving the competencies necessary for their public health training program. This balance is possible if fueled by curiosity, adaptability, cultural humility, and determination to improving community health. Notably, commitment to hours outside the traditional classroom by students and instructors, is vital. These hours to coach and model students at community sites, and the flexible and adaptable stance of instructors to adjust to the realities of community partner organizations is necessary for attaining the goals of the LC.

The evaluation presented has several strengths. It provides an assessment of all LC cohorts over time, preliminary evidence of the value of the experience after graduation, as well as the perspective of community-based organizations involved in the LC. In addition, this longer-term evaluation with mixed methods supports the value of this pedagogical approach and expands with evidence beyond other models described. However, this evaluation has several limitations. First, it is limited by its sample size. More than 50% of LC alumni responded to the survey, representing just 30 students, though representing different cohorts of the LC since its inception. Also, only 3 community partners responded to the survey. Second, the quantitative alumni survey items were developed to assess the competencies that are supported by the LC by the authors, but these items are not part of a standardized assessment, limiting the validity of this assessment. Nonetheless, the contribution of the survey is provided by the comments that alumni provided, the qualitative component of this evaluation. Third, this examination relied on self-reported experiences. Further examinations could rely on more objective assessment of the domains addressed by the LC, as well as controlled comparison to other student groups that do not receive the same learning experience. While these approaches could control for other confounders, they will not definitively control for bias resulting from non-randomized designs. Fourth, while responses are anonymous, these might be limited by social desirability and recall bias. Fifth, while some of the components of the LC are common to other practice-based approaches, generalizability is limited to other student populations or other teaching approaches.

Public health education is being challenged to innovate and develop students who will become change agents and who can combat the inequities in health being experienced across the globe today. The LC appears to be a promising model for applied and science-based public health education and community intervention. It provides students with opportunities to integrate, apply, and strengthen cross-cutting public health skills like community engagement, collaboration, and needs assessment, in a supportive environment, while enhancing the sustainability of student and faculty’s community public health work.

## Data availability statement

The raw data supporting the conclusions of this article will be made available by the authors, without undue reservation.

## Author contributions

VEH: Conceptualization, Formal analysis, Methodology, Project administration, Supervision, Writing – original draft, Writing – review & editing. TP: Conceptualization, Project administration, Writing – original draft, Writing – review & editing. JK: Writing – review & editing, Conceptualization. RDS: Writing – review & editing, Formal analysis, Methodology, Writing – original draft. STG: Formal analysis, Writing – review & editing.
